# Development of significant disease in a cohort of patients with non-specific enteritis on capsule endoscopy: clinical suspicion and a high base line Lewis score are predictive of Crohn’s disease

**DOI:** 10.1186/s12876-020-01486-7

**Published:** 2020-10-15

**Authors:** Sandeep Sihag, Brandon Tan, Serhiy Semenov, Mohd Syafiq Ismail, Barbara Ryan, Anthony O’Connor, Niall Breslin, Rita Douglas, Deirdre McNamara

**Affiliations:** 1 Department of Gastroenterology, Tallaght University Hospital, Dublin, Ireland; 2 TAGG Research Centre, Trinity Centre, Tallaght Hospital, Dublin 24, Ireland

**Keywords:** Capsule endoscopy, Crohn’s disease, Enteritis

## Abstract

**Background:**

As with isolated ileitis the findings of nonspecific small bowel enteritis (NSE) on capsule endoscopy (CE) poses a clinical challenge. There is lack of available evidence to help clinicians to predict significant disease and long-term prognosis.

**Aim:**

To define the natural history of NSE in an Irish cohort.

**Methods:**

Patients with a finding of NSE were identified from a database. Subsequent investigations, treatments and diagnosis were recorded. Patients were grouped based on ultimate diagnosis: Crohn’s disease (CD), Irritable bowel syndrome (IBS), NSAIDs enteritis (NSAIDs), persistent NSE and no significant disease (NAD).

**Results:**

88 patients, 46 (52%) male, mean age 52 ± 17.8 years were included with a mean follow up of 23 ± months. The ultimate diagnoses were NAD = 43 (49%), CD = 17 (19%), IBS = 14 (16%), NSAIDs = 12 (14%) and persistent NSE = 2 (2%). Significantly, more patients diagnosed with CD on follow up were referred with suspected CD. CD = 14/17 (82%) vs 13/57 (23%), *p* < 0.001. While a diagnosis of CD was associated with a positive baseline Lewis score (> 135); 11/17 (65%) CD versus 16/ 71 (23%). Female gender was associated with an ultimate diagnosis of IBS (OR 5, *p* < 0.02). Older age was associated with NSAIDs enteritis, while more subjects without significant gastrointestinal disease were anemic on presentation.

**Conclusion:**

The majority (49%) of NSE patients do not develop significant small bowel disease. CD occurred in 19% of NSE patients on follow up. Clinical suspicion and capsule severity are predictive of Crohn’s disease on initial CE.

## Background

Capsule endoscopy is now widely accepted and is a routine diagnostic tool for a variety of small bowel symptoms and conditions, with European clinical, technical and quality guidelines available to enhance capsule practice [1–3]. While capsule endoscopy is a relatively simple and non-invasive procedure, reading of videos and interpretation of findings is more challenging and requires adequate training [4]. In particular, allocating appropriate clinical relevance to identified small bowel lesions can be problematic. In cases performed for suspected small bowel bleeding the Saurin classification is recommended and routinely employed too assign a significance level and has been shown to be effective [5]. Similarly, in patients with known Crohn’s disease both the Capsule Endoscopy Crohn’s disease Activity Index (CECDAI) and Lewis Scores are ways to grade activity [6, 7]. However, neither score has been validated as a diagnostic tool for Crohn’s disease and cannot easily identify clinically significant small bowel inflammatory lesions. Previous consensus statements have highlighted the lack of a gold standard diagnostic test for Crohn’s disease and suggested findings need to be interpreted with a full knowledge of the subject’s clinical history [8]. The presence of classical large/deep ulcers, stenosis and mucosal inflammation combined with a relevant clinical history (NSAID use/Radiation exposure) can usually accurately predict disease in severe cases. While the non-specific nature of lesser lesions, scattered small erosions/aphthous ulcers with minimal or mild inflammation, without a supportive medical history poses a diagnostic difficulty. Particularly as histological correlation, although often recommended, is not always readily available. Even if device assisted enteroscopy is possible, or repeat ileo-colonoscopy is able to reach the identified inflammatory lesions, for mild cases of enteritis with a low clinical suspicion of significant disease, further endoscopy may not be deemed appropriate. As a result, non-specific enteritis, not reaching a diagnostic threshold for disease is a relatively frequent finding in capsule endoscopy, its relevance and clinical importance is poorly understood. It has long been recognized that not all inflammatory ileal lesions represent Crohn’s disease and there is a broad differential diagnosis [9]. Several studies have reported the natural history of isolated ileitis identified in both symptomatic and asymptomatic cohorts found on routine ileo-colonoscopy [10–15]. While the majority report low rates of significant disease on follow up, up to a quarter were ultimately diagnosed with Crohn’s disease. Currently the natural history of non- specific enteritis diagnosed on capsule endoscopy and the incidence of clinically relevant disease is unknown. Capsule endoscopy is reserved for cases of suspected small bowel disease, following negative standard bi-directional endoscopy, in the case of bleeding and after negative ileo-colonoscopy and cross-sectional imaging in cases of suspected Crohn’s disease. In addition, capsule endoscopy enables detection of inflammatory lesions throughout the small bowel. As such extrapolating data from ileo-colonoscopy isolated active ileitis studies may miss-interpret the relevance of CE disease. Identification of at risk characteristics in non-specific enteritis cases enabling capsule readers to safely predict clinical relevance and direct and target further investigations are needed.

## Aim

To define the natural history of non-specific enteritis in a capsule endoscopy cohort.

## Methods

Following approval as a service evaluation from our Hospital Review Board, subjects with a diagnosis of non-specific enteritis were identified from a capsule endoscopy database from 2014 to 2019. Patients with known Crohn’s disease, and enteritis with a definitive diagnosis (known regular NSAID exposure, diaphragm disease, radiation enteritis, combined variable immuno deficiency syndrome associated enteritis, cryptogenic multi focal ulcerous stenosing enteritis, lupus enteritis, chemotherapy associated enteritis, CMV or other infective enteritis) and those with abnormal radiology or clinical findings highly suggestive of Crohn’s and those with < 3 months follow up or from external institutions were excluded. All capsule studies were performed after a negative baseline bidirectional endoscopy. Capsule studies were performed as standard after preparation with either a PillCam SB2 or 3 (Medtronic, Minneapolis, USA) and read by experienced capsule readers using Rapid Reader Software. All patients were requested to discontinue Iron and Aspirin for 1 week prior to the procedure. All reports were reviewed and approved by our institutions capsule review board. Basic demographics, indication for capsule and initial findings were all documented from the baseline capsule report.

The clinical records of non-specific enteritis cases were reviewed and subsequent additional investigations (endoscopies, imaging, biomarkers and blood tests), treatments and diagnosis were recorded. Patients were subsequently grouped based on ultimate clinical diagnosis as follows: Crohn’s disease (CD), Irritable bowel syndrome (IBS), NSAIDs enteritis (NSAIDs), no significant gastrointestinal disease (NAD), and persistent non-specific enteritis (NSE). An ultimate diagnosis of Crohn’s disease was established based on the WHO 2015 Diagnostic Criteria including characteristic clinical features, endoscopic and radiology features and histology. Patients were diagnosed with IBS on follow up if there was an absence of definitive diagnostic criteria for another diagnosis and/or the resolution of enteritis on additional small bowel investigations, were available and the presence of typical Rome III Criteria symptoms. Clinical and demographic parameters were documented and compared between the groups using a student *t*-test for continuous data and Chi squared for categorical data and *p* < 0.05 were considered significant.

## Results

In all 326 of 2500 (13%) capsules reviewed had a finding of enteritis. Of these 169 were external patients with no available follow up and 69 had known Crohn’s disease and were excluded. In all 88 (27%) patients were included in our analysis, 46 (52%) male, mean age 52 ± 17.8 years, with a mean follow up of 23 ± 19 months (range 3–88 months). Of these 85 (97%) were complete studies and image quality was reported as good or adequate in 86 (98%). As expected in a cohort with NSE, not meeting a diagnostic threshold for Crohn’s disease there were no strictures and no retained capsules.

The indications given for capsule endoscopy were suspected Crohn’s disease in 37 (42%), obscure occult bleeding in 23 (26%), obscure overt bleeding in 15 (17%) and other in 13 (15%) subjects respectively. In all, 105 additional endoscopies were performed in 70 (80%) subjects during the follow up period, including an ileo-colonoscopy and device assisted enteroscopy in 40 (45%) and 30 (34%) subjects respectively. While 14 (16%) had a repeat capsule endoscopy and 18 (20%) additional small bowel imaging. In addition, during follow up a C-reactive Protein (CRP) was available in 61 (69%), a Fecal Calprotectin (FC) in 28 (32%), while a hemoglobin was documented in 53 (60%) subjects.

The ultimate clinical diagnoses on follow up of our cohort were Crohn’s disease in 17 (19%), NSAID related enteritis in 12 (14%), irritable bowel syndrome in 14 (16%), persistent non-specific enteritis in 2 (2%) and no significant gastrointestinal disease in 43 (49%) subjects (Table 1).

**Table 1:** Study population

Study population n = 88	
Mean age in years	52 ± 17.8
Male gender n (%)	46 (52%)
Mean follow up in months	23 ± 19
Indication for capsule n (%)	
Suspected Crohn’s disease	37 (42%)
Obscure occult bleeding	21 (24%)
Obscure overt bleeding	14 (16%)
Other	16 (18%)
Ultimate diagnosis n (%)	
Crohn’s disease	17 (19%)
Irritable bowel syndrome	14 (16%)
NSAID enteritis	12 (14%)
Persistent non-specific enteritis	2 (2%)
No significant GI disease	43 (49%)

Significantly more patients diagnosed with CD and IBS on follow up were referred with suspected CD; CD = 14/17 (82%), (OR 9, *p* < 0.009, 95% CI 2.54–37.58) and IBS = 10/14 (71%) (OR 3, 95% CI 0.97–12.09, *p* < 0.05) vs 13/57 (23%). In addition, a diagnosis of CD was associated with a positive baseline Lewis score (> 135); 11/17 (65%) CD versus 16/ 71 (23%), OR 6, 95% CI 2.01–19.70, *p* = 0.002. The distribution of the inflammation did not vary with ultimate diagnosis. Similarly, neither a raised CRP (> 5 mg/L) nor a raised FC (> 50 μg/g) was predictive of outcome. Of note no patient had a FC higher than 250 μg/g. Female gender was associated with an ultimate diagnosis of IBS (OR 5, 95% CI 1.30–19.76, *p* < 0.02). While older age (64.1 vs 50.3 years, *p* < 0.01, 95% CI − 24.5 to − 3.11) was associated with an ultimate diagnosis of NSAIDs enteritis. Of interest anemia 9/23 (39%) versus 2/30 (7%) (Hb < 11.5 g/dl for women and < 12.6 g/dl for men) and lower mean baseline Hemoglobin (11.3 g/dl vs 13.6 g/dl, *p* < 0.0004, 95% CI − 3.51 to − 1.08) was present in the cohort found to have no significant gastrointestinal disease on follow up (Table 2).

**Table 2:** Study characteristics according to ultimate diagnosis

Diagnosis	Crohn’s disease	NSAID enteritis	Irritable bowel syndrome	Persistent non-specific enteritis	No significant disease
Number	17	12	14	2	43
Mean age (Years)	48 ± 10	64 ± 14^a^	42 ± 15	28	52 ± 19
Male gender N (%)	7 (41%)	5 (42%)	3 (21%)^a^	2 (100%)	29 (67%)
Lewis score > 135 n (%)	11 (65%)^a^	5 (42%)	6 (43%)	1 (50%)	4 (9%)
Proximal disease N (%)	4 (24%)	2 (17%)	3 (21%)	1 (50%)	14 (32%)
Fecal Calprotectin > 50 μg/g N (%)	4 (57%)	2 (100%)	5 (55%)	0	3 (33%)
CRP < 5 mg/L N (%)	5 (36%)	3 (36%)	3 (21%)	0	5 (21%)
Mean Hemoglobin g/dl	14 ± 2.5	12.8 ± 2.3	13.8 ± 1.2	13.8	11.3 ± 2.4

^a^Statistically significant difference

## Discussion

Our novel study suggests non-specific enteritis is a relatively frequent finding in capsule endoscopy practice, in our cohort 13% of capsule studies had inflammatory lesions not meeting a diagnostic threshold. While the majority settled on follow up 49%, almost 1:5 were ultimately diagnosed with Crohn’s disease. Figures which are in keeping with follow up data of patients with acute isolated active ileitis, even though all our subjects had a negative ileo-colonoscopy prior to capsule endoscopy. The presence of skip lesions are characteristic of Crohn’s disease and may explain the initial negative ileo-colonoscopy, as could the variation in mucosal lesions over time [16].

An indication of suspected Crohn’s disease for capsule endoscopy was strongly associated with subsequent Crohn’s on follow up, OR 9. Not surprisingly, patients with a subsequent diagnosis of IBS were also more likely to have been referred with suspected Crohn’s disease. However, CD patients alone were more likely to have an abnormal/elevated base line Lewis Score, OR 6. Of note traditional IBD associated biomarkers, CRP and FC did not predict small bowel CD, nor did the extent or distribution of inflammatory lesions. This finding is in keeping with previous longitudinal studies of isolated terminal ileitis on ileo-colonoscopy which demonstrated a positive association with symptoms and subsequent Crohn’s development and a lack of predictive value for either biomarkers, baseline histology or family history [12, 13, 15]. Unfortunately, the Lewis Score alone is not a sensitive test for CD. The combination of both indication and Lewis Score may be helpful in predicting subsequent CD in NSE patients and warrants further study.

The finding that more subjects without significant gastrointestinal disease on follow up were anemic may simply reflect the fact that the largest indication for CE in our practice is anemia with or without overt gastrointestinal bleeding. None had concomitant vascular small bowel lesions.

Of interest is the significant number of patients in our cohort who were ultimately diagnosed with NSAID related enteritis (14%). While our unit’s patient information and advice leaflet specifically requests patients to avoid NSAID’s for 6 weeks prior to their capsule endoscopy, and patient reported current medications use is documented on the day of their procedure, the video readers were not always aware of the possibility of NSAID enteritis as a diagnosis. Only on subsequent review with a targeted medication history, including over the counter formulations, was the diagnosis established. This highlights the already identified need to interpret inflammatory lesions with a full knowledge of the patient’s clinical history, and the importance of considering possible NSAID exposure in cases of NSE and reviewing medication exposure on follow up [8]. Of interest is the finding that NSAID related disease on follow up was associated with older age. As such this cohort were more likely to have increased numbers of comorbidities and poly-pharmacy may have contributed to the problem.

Our study has several limitations. Firstly the majority of cases, 52% (169/326) with enteritis either Crohn’s or NSE, were performed on patients from outside our institution with no available follow up data. Despite this our cohort represents the largest NSE capsule study (n = 88), with a median follow up of almost 2 years. In addition, with the ready availability of device assisted enteroscopy and capsule endoscopy at our institution, a significant proportion of our population underwent additional endoscopic investigations as well as gastroenterology clinical review adding weight to our findings. On the other hand only a small proportion underwent subsequent dedicated small bowel imaging (20%). It is likely that many, particularly those with suspected Crohn’s disease, already had negative imaging, in keeping with CE referral practice and local guidelines. While there is evidence to link terminal ileal narrowing on CT scans with a subsequent diagnosis of Crohn’s disease on ileocolonoscopy [14], evidence from a recent prospective study of patients with abnormal small bowel MRE findings with a negative initial ileo-colonoscopy suggested few develop significant disease (12%) [10]. As such the added value of subsequent imaging may be low.

## Conclusion

Nonspecific enteritis on capsule endoscopy is a not infrequent finding and represents a diagnostic challenge. Only a minority go on to a diagnosis of Crohn’s disease. Clinical suspicion of Crohn’s and a high baseline Lewis score are predictive of Crohn’s disease and may be useful indicators of the need for closer follow up and additional investigations.

## Data Availability

The datasets generated and/or analysed during the current study are not publicly available in keeping with GDPR guidelines on management of patient information but are available from the corresponding author on reasonable request.

## Abbreviations

NSE: Nonspecific small bowel enteritis
CE: Capsule endoscopy
CD: Crohn’s disease
IBS: Irritable bowel syndrome
NSAIDs: NSAIDs enteritis
NAD: No significant disease
CECDAI: Capsule Endoscopy Crohn’s disease Activity Index
CRP: C-reactive Protein
FC: Fecal Calprotectin
